# Simultaneous Voltammetric Determination of Acetaminophen, Ascorbic Acid and Uric Acid by Use of Integrated Array of Screen-Printed Electrodes and Chemometric Tools

**DOI:** 10.3390/s19153286

**Published:** 2019-07-26

**Authors:** Dionisia Ortiz-Aguayo, Marta Bonet-San-Emeterio, Manel del Valle

**Affiliations:** Sensors and Biosensors Group, Department of Chemistry, Universitat Autònoma de Barcelona, 08193 Bellaterra, Spain

**Keywords:** electronic tongue, modifiers, acetaminophen, ascorbic acid, uric acid, partial least squares regression

## Abstract

In the present work, ternary mixtures of Acetaminophen, Ascorbic acid and Uric acid were resolved using the Electronic tongue (ET) principle and Cyclic voltammetry (CV) technique. The screen-printed integrated electrode array having differentiated response for the three oxidizable compounds was formed by Graphite, Prussian blue (PB), Cobalt (II) phthalocyanine (CoPc) and Copper oxide (II) (CuO) ink-modified carbon electrodes. A set of samples, ranging from 0 to 500 µmol·L^−1^, was prepared, using a tilted (3^3^) factorial design in order to build the quantitative response model. Subsequently, the model performance was evaluated with an external subset of samples defined randomly along the experimental domain. Partial Least Squares Regression (PLS) was employed to construct the quantitative model. Finally, the model successfully predicted the concentration of the three compounds with a normalized root mean square error (NRMSE) of 1.00 and 0.99 for the training and test subsets, respectively, and R^2^ ≥ 0.762 for the obtained vs. expected comparison graphs. In this way, a screen-printed integrated electrode platform can be successfully used for voltammetric ET applications.

## 1. Introduction

**Figure 1:**
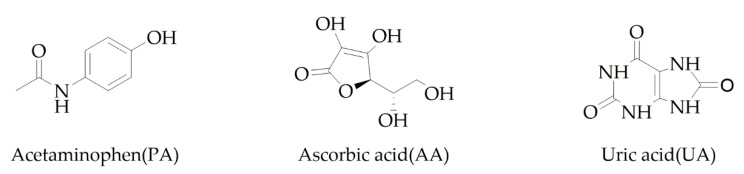
Chemical structure of three studied compounds in this report (Acetaminophen, Ascorbic acid and Uric acid).

Acetaminophen, Ascorbic acid and Uric acid (Figure 1) play an important role in humans’ life. Acetaminophen (N-acetyl-p-aminophenol or paracetamol (PA)) is an antipyretic and analgesic drug commonly used against arthritis, headache, muscle aches, menstrual cramps and fevers [1]. A high amount of PA can cause the accumulation of toxic metabolites, leading to severe and sometimes fatal hepatotoxicity and nephrotoxicity [2]. Ascorbic acid (AA) is a vitamin commonly present in many biological systems and in multivitamin formulations. It is widely employed to provide an adequate dietary intake and as an antioxidant [3]. Its excessive dose may cause headache, trouble sleeping, gastrointestinal discomfort and flushing of the skin [4]. Uric acid (UA) is the primary product of purine metabolism [5]. Continuous monitoring of UA in the body fluid is essential since its abnormal concentration levels lead to several diseases, such as hyperuricemia and gout [6]. Other diseases, such as leukemia and pneumonia are also associated with enhanced urate levels.

Several analytical methods for individual or simultaneous determination of PA, AA and UA have been reported in the literature such as spectrofluorometry [7,8], spectrophotometry [9,10], chromatography [11,12], and capillary zone electrophoresis [13,14]. The problem is that these methods can be expensive and need complex procedures. For these reasons, the development of rapid, cheap and effective determination procedures is needed. One proposal to overcome this can be the development of electrochemical sensors [15]. This kind of devices provide some advantages, such as, low detection limits, wide linear response range, good stability and reproducibility.

However, certain difficulties arise when the simultaneous determination of these three compounds is attempted. The oxidation peaks of PA, AA and UA are almost overlapping on traditional electrodes [16], which make their simultaneous determination highly difficult. One solution to solve the main drawback is the use of methods based on modified electrodes, which have fascinated many researchers due to their simplicity, high sensitivity, and low cost. In addition, this strategy allows some improvement based on electrocatalysis, liberation from surface fouling and prevention of undesirable reactions competing kinetically with the desired electrode process [17].

Modified electrodes [18] can be prepared by several different techniques based on adsorbing, attaching specific molecules (e.g., peptides [19] or complexing agents [20,21]) to the surface by self-assembled monolayer [22], coating and entrapment, e.g., is the form of conductive ink [23]. The last strategy has become interesting for electrochemists in recent times, because this deliberate and controlled modification of the electrode surface can produce new surfaces with interesting properties employed for new devices and applications in electrochemistry.

Nature has developed and optimized an impressive variety of sensing systems used for navigation, spatial orientation, prey detection, object inspection, peer interaction, etc. which provide technologist with inspiring ideas for new concepts for sensors or improvements within the field [24,25]. Illustrating examples in chemical sensing is the development of electronic noses (EN) and electronic tongues (ET), both sharing the concept of preferring a number of sensors (a sensor array) with broad selectivity pattern, instead of a single, highly selective sensor. The use of this number of receptors in a combinatorial way is what permits to the animal senses to be effective in detecting thousands of different compounds or situations. In the field of chemical analysis, the main bioinspired systems take after three mammal senses: smell, taste and sight. Therefore, there have been reported electronic noses (EN) [26], eyes (EE) and tongues (ET) [27]. From these principles, the EN, formed by an array of sensors with slightly different response to generic compounds has been used for analysis in the gas phase and stands out its closeness to artificial olfaction. In the case of EE, there are also interesting advances reported in the literature. An example is the development of a bioinspired electronic white cane for blind people using whiskers multiple sensor principle for short-range navigation and exploration [28].

Similar to the EN is the ET that, according to IUPAC [29], is defined as is a multisensor system, which consists of a number of low-selective sensors and uses advanced mathematical procedures for signal processing based on pattern recognition and/or multivariate data analysis. This analytical system applied to liquid analysis allows the generation of multidimensional information in combination with chemometric processing, which allows extracting the maximum chemical information from these complex data.

In this way, biomimetic systems, in opposition of classical approaches, use the combination of low selective and/or cross-responsive sensors to obtain rich and complementary analytical information. Next, this complex, multi-dimensional information needs to be processed with proper data treatment tools, which is accomplished with chemometrics. This coupling has been declared one of the ways of progress in developing new sensing schemes [30]. There are different data processing tools depending on the final application needed. If this is a qualitative goal, PCA is a suitable linear visualization/pattern recognition method. This tool allows the reduction of the dimensionality of a multivariate problem and facilitates the visualization of different categories of the multivariate profiles by remarking similarities and differences between sample clusters. When the purpose is quantitative, different tools are available, given the numeric information is the end result. Some of these are Principal Component Regression (PCR), which departs from a first PCA transformation to build a multivariate regression, Partial Least Squares Regression (PLS), or Artificial Neural Networks (ANNs) [31].

In the present work, an eight sensor integrated array of screen-printed electrodes has been developed in base of a multiple screen-printed carbon electrode (SPCE) platform. The voltammetric array, consisting of Graphite/SPCE-Ink, Prussian blue/SPCE-Ink, Cobalt (II) phthalocyanine/SPCE-Ink and Copper oxide (II)/SPCE-Ink was employed for the simultaneous determination of the three aforementioned compounds (PA, AA and UA) by using the Cyclic voltammetry (CV) technique. This represents an example of resolving a mixture where heavily interfering signals are generated and resolving its components is difficulted. In other words, it is shown how to detect simultaneously the different analytes in presence of their interferents, which redox signals overlap. For showing these aspects, firstly, the behavior of the sensors was evaluated separately for each compound; secondly, peak current responses showed that all sensors had differentiated response for the three oxidizable compounds of clinical interest. Finally, a response model was developed to determine mixtures of PA, AA and UA at the µmol·L^−1^ level.

## 2. Materials and Methods

### 2.1. Chemicals and Reagents

All solutions were made up using sterilized Milli-Q water (Millipore, Billerica, MA, USA). Cobalt (II) phthalocyanine (CoPc), Copper (II) oxide (CuO) nanopowder (<50 nm), Polypyrrole doped (PP) and Palladium, powder submicron 99.9+% (Pd), which were used as modifiers, were purchased from Sigma-Aldrich (St. Louis, MO, USA). Prussian blue was from Acros Organics (Geel, Belgium). The preparation of the ink composite was done using mesitylene and polystyrene, obtained from Sigma-Aldrich (St. Louis, MO, USA). Graphite powder (particle size < 50 µm) was received from BDH (BDH Laboratory Supplies, Poole, UK). Potassium chloride was purchased from Merck (Darmstadt, Germany).

Acetaminophen (PA), Ascorbic acid (AA), Uric acid (UA) and hydrogen peroxide (H_2_O_2_) solution were purchased from Sigma-Aldrich (St. Louis, MO, USA).

All the measurements were carried out using 50 mM phosphate buffer (PBS) solution and 0.1 M KCl solution at pH 7.

### 2.2. Electronic Tongue

The voltammetric ET was formed by an integrated array of eight screen-printed electrodes as working electrodes (8W110 Electrodes, ceramic substrate: 50 × 27 × 1 mm. and electric contacts composed of silver) from DropSens (Oviedo, Spain). The electrochemical cell consisted on: 8 working electrode (carbon, 2.95 mm diameter), auxiliary electrode (carbon) and pseudo reference electrode (Silver) [32].

Electrochemical measurements were performed at room temperature (25 °C), using a portable Multi Potentiostat/Galvanostat µStat 8000 from DropSens controlled through its Dropview Multichannel 5.5 software package. A complete Cyclic voltammogram was recorded for each sample and for each electrode by cyclic the potential between −1.5 and +1.5 V with a step potential of 9 mV and a scan rate of 50 mV·s^−1^.

### 2.3. Modification of the Electrode Surface

**Figure 2:**
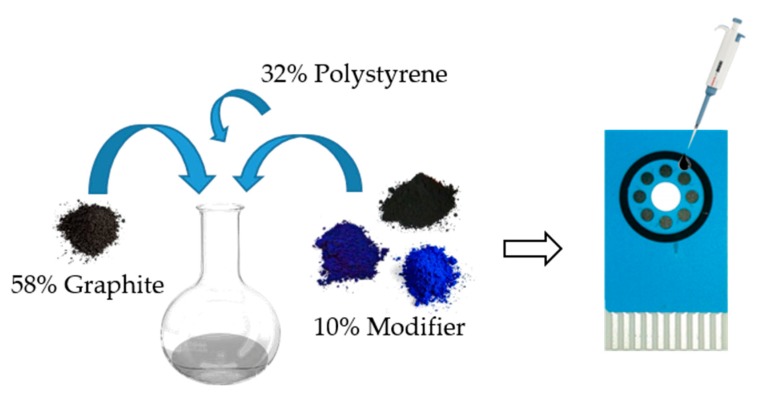
Scheme of the experimental procedure for the electrode surface modification. Firstly, an ink-like solution was prepared incorporating the corresponding modifier. Then, 5 µL was dropped on the electrode surface and dried a 40 °C. An activation step is done with hydrogen peroxide to finally record the voltammetric signal.

**Figure 3:**
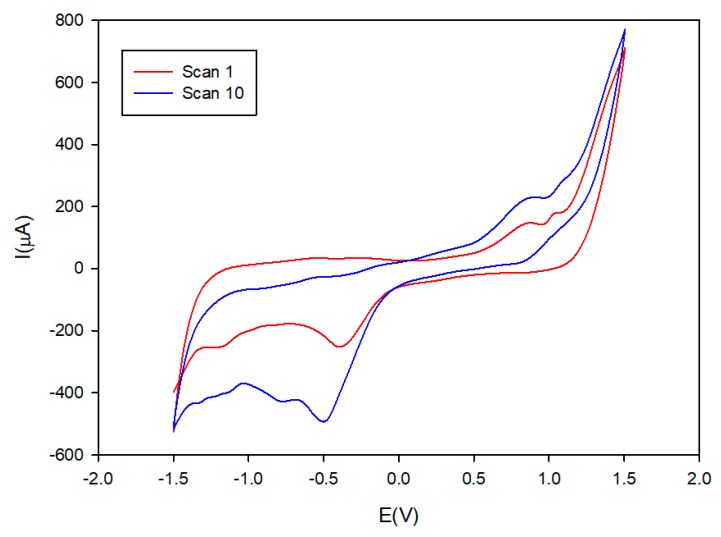
Comparison of the Cobalt (II) phthalocyanine/SPCE-Ink before (scan 1) and after (scan 10) the activation. Electrochemical activation consists of 10 repetitive voltammetric cycles at 50 mV·s^−1^ between 1.5 and −1.5 V using 10 mM H_2_O_2_ in phosphate buffer (pH 7). After activation, electrodes were rinsed with deionized water and dried in air.

The nanomaterial SPCE/modifier was produced in the form of a conductive ink-like composite. The corresponding modifier, graphite and polystyrene were thoroughly mixed with mesitylene for 2 h (Figure 2) using a magnetic stirrer. After that, 2 min of sonication was performed in order to obtain a medium thick solution. The ink-like composite was dropped 5 µL onto the surface of a screen-printed carbon electrodes (SPCE) and dried at 40 °C for at least 1 h in order to remove the solvent. Once the sensor was prepared, the next step is an activation [33,34] in order to enhance sensing performances of modified ink (Figure 3 displays the typical gain achieved after activation). Electrochemical activation consisted of 10 repetitive voltammetric cycles at 50 mV·s^−1^ between 1.5 and −1.5 V using 10 mM H_2_O_2_ in phosphate buffer (pH 7). After activation, electrodes were rinsed with deionized water and dried in air.

### 2.4. Characterization by Scanning Electron Microscopy

The morphological characterization of the modified screen-printed electrode was performed by Scanning Electron Microscopy (SEM). A scanning electron microscope with field emission gun (FEG-SEM) of Zeiss, model MERLIN SM0087 was used.

### 2.5. Sample Preparation

According to the European Pharmacopoeia [35] the size of the data set needed for building the calibration is dependent on interfering properties and the number of analytes that needs to be handled in the model. In the majority of the cases, the size of the learning data set for calibration needs to be large when the interfering variations are acquired randomly. However, when the major interferences can be controlled they can be varied according to a statistical experimental design.

**Figure 4:**
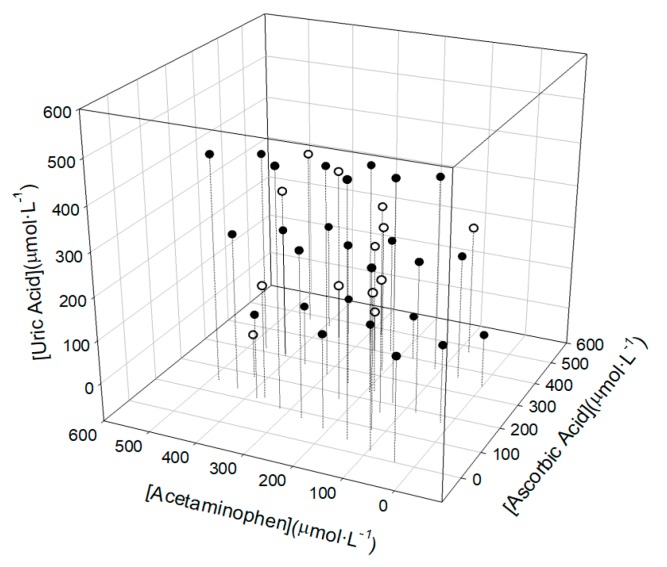
Representation of the tilted factorial experimental design employed (3^3^) where it can be observed how the test samples (in white) are distributed covering all the space of the training samples (in black).

In this case, the second option was accomplished using a tilted factorial experimental design [36] 3^3^ (27 samples) for the train subset. This tilted model consisted of a factorial design with a 45° rotation in each axis. With this approach it is possible to avoid the repetition of numeric values. Meanwhile, the validation of the constructed model was done with an external test set (12 samples), these were distributed randomly within the experimental domain (0 to 500 µmol·L^−1^) for each compound (Figure 4).

Samples were prepared in buffer solution (50 mM phosphate buffer solution at pH 7 containing 0.1 M KCl). Fresh stock solutions of pharmaceutical compounds were prepared the same day of the measurements, in order to avoid/reduce the day-to-day variability.

### 2.6. Data Processing

The statistical treatment and data analysis were performed using routines developed by the authors using MATLAB R2017a (MathWorks, Natick, MA, USA); in particular, the functionalities “plsregress” from the Statistics and Machine Learning Toolbox, was the one employed for the response model; the web page Clustvis [37] was the one used for PCA calculation; Sigmaplot (Systat Software Inc., San Jose, CA, USA) was used to graphically represent and analyze the results.

## 3. Results and Discussion

### 3.1. Characterization of the Surface

**Figure 5:**
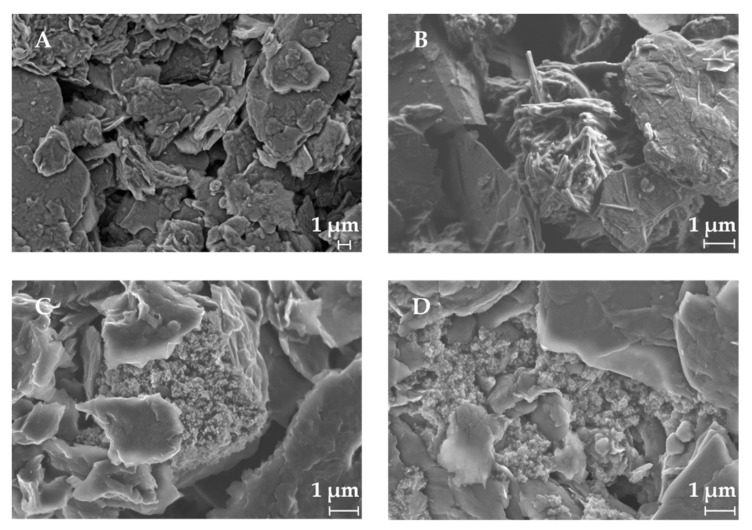
Scanning Electron Microscopy (SEM) characterization of (**A**) Graphite/SPCE-Ink, (**B**) Cobalt (II) phthalocyanine/SPCE-Ink, (**C**) Copper oxide (II)/SPCE-Ink and (**D**) Prussian blue/SPCE-Ink.

A SEM characterization was performed in order to investigate the spatial distribution of the ink-nanoparticles and to verify if the particles were all on the external surface or in the inner layers. As can be observed in Figure 5, the different modifiers are distributed quasi-homogeneous between the graphite layers. The size of the nanoparticles is below 1 µm.

### 3.2. Voltammetric Array Response

**Figure 6:**
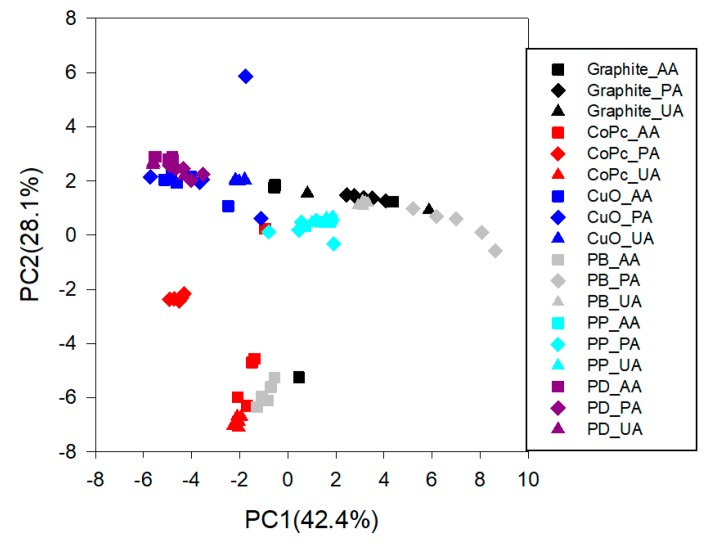
Score plot of the two components obtained after Principal Component Analysis (PCA) analysis. Five replicates for each sensor were done determining the three compounds of interest: Acetaminophen, Ascorbic acid and Uric acid.

The four aforementioned modifications used to perform this work, were selected among six modifications candidates (Graphite, Cobalt(II) phthalocyanine, Copper oxide (II), Prussian blue, Polypyrrole doped and Palladium) to construct the sensor array. This selection facilitates the modification in form of an ink. In addition, these nanomaterials are the ones with extended experience in the laboratory (although used in a different electrode format, the epoxy-graphite composites [38]). The behaviors of these sensors for each compound were evaluated individually. Once the voltammograms were collected, a preliminary qualitative analysis was performed in order to evaluate the complementary of the sensors. The chemometric tool used was PCA. The information collected in this case to perform the PCA calculation was the unfolded data. If electrodes are redundant they would appear superimposed in the PCA graph, while a different response will manifest in their separation. As can be seen in Figure 6, each sensor showed performance in different regions. This fact accomplishes a relevant role in the execution of the electronic tongue, justifying the good contribution of the four prepared electrodes in the sensor array. In addition, this analysis made us discard from the sensor array system the Palladium and Polypyrrole sensors, because they were not able to provide distinction between the different substances (Figure 6). According to these criteria, the other modifiers could be classified as good candidates because they provided a wide range of variability between the substances. In addition, the vast majority of the signals were far away from zero, meaning they provide useful information to the system.

After this pre-selection step, voltammograms for each of the selected electrodes towards individual compounds were secondly evaluated. Two scans were performed choosing the second one to represent the voltammetric response.

**Figure 7:**
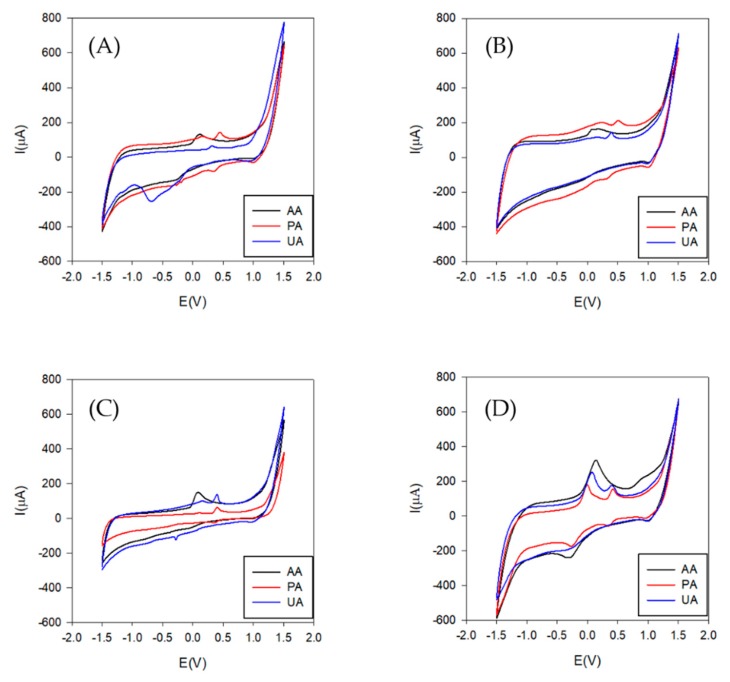
Voltammetric response for Acetaminophen (PA), Ascorbic acid (AA) and Uric acid (UA) using the four finally selected inks. (**A**) Cobalt (II) phthalocyanine/SPCE-Ink; (**B**) Prussian blue/SPCE-Ink; (**C**) Graphite/SPCE-Ink; (**D**) Copper oxide (II)/SPCE-Ink. The range of potential was from −1.5 to 1.5 V. The scan rate was 50 mV·s^−1^ and step rate of 9 mV. A 300 µmol·L^−1^ individual solution was employed for the four modified screen-printed electrodes.

Therefore, following the conditions described in Section 2.1, a stock solution of 300 µmol·L^−1^ of these compounds was evaluated. As can be observed in Figure 7, slightly different signals were obtained for the different compounds of interest, a necessary requirement for the performance of the electronic tongue.

### 3.3. Characterization of the Modified Integrated Screen-Printed Electrodes

#### 3.3.1. Calibration Curves

To evaluate the behavior of each sensor for each compound separately, some calibrations curves were performed using Cyclic voltammetry (CV) technique representing the peak height which corresponds to the maximum of the oxidation signal. This kind of experiment is important to determine the linear range and the maximum concentration of each compound for the final experiment (electronic tongue).

**Figure 8:**
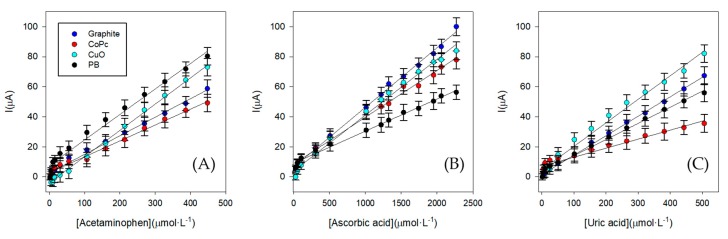
Calibration curves for three replicates (n = 3) to determine the concentration working range for the three compounds under study for the four modified screen-printed electrodes. (**A**) Acetaminophen, (**B**) Ascorbic acid, (**C**) Uric acid.

As can be observed in Figure 8, the studied ranges were linear for all the compounds. Therefore, for the ET development the concentration working range was from 0 to 500 µmol·L^−1^ for Acetaminophen, Ascorbic acid and Uric acid. The linear relationship and the calibration equations for each sensor are represented in Table 1.

#### 3.3.2. Stability and Reproducibility Studies

After the calibration characterization, a stability and reproducibility study was done in order to verify that the sensors were capable of supporting the number of measurements necessary for the Electronic tongue (ET) development. The procedure used to analyze the durability/stability of the sensors consisted on measuring a stock solution of Acetaminophen (165 µmol·L^−1^) 30 times. A blank, in PBS solution, was inserted between each measurement to evaluate if the system was presenting fouling effect.

In all cases, the four sensors showed stable responses with Relative Standard Deviation (%RSD) of 3.9%, 6.3%, 5.5% and 8.2% for Graphite, Copper oxide (II), Prussian blue and Cobalt (II) phthalocyanine electrodes, respectively. No fouling effect was either observed in this study, when examining any trend among the blanks.

The next step was to study the reproducibility of construction of the ink-modified SPCE sensor. The experiment was done preparing four sensors by triplicate (n = 3) and measuring consecutively with an acetaminophen stock solution. The results for each sensor present a good reproducibility, showing the best for the Prussian blue with a relative standard deviation (RSD) of 0.8% (Table 2).

### 3.4. Qualitative Analysis: Principal Component Analysis (PCA)

**Figure 9:**
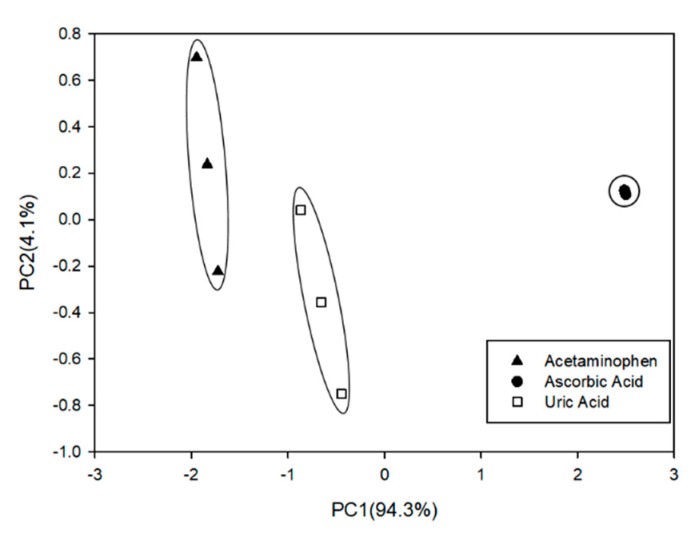
Score plot of the two components obtained after PCA analysis. The PCA shows three clusters across the PC1 (94.3%). These clusters correspond to the studied compounds for n = 3 replicates. These results were obtained using the sensor array chosen.

Once the characterization was accomplished, a PCA was performed in order to evaluate the discrimination power of the sensors (Figure 9). The information collected in this case to perform the PCA calculation was the sensitivity of the calibration curves. Once it was confirmed that the different electrodes presented a differentiated electrochemical behavior towards the different compounds under study, allowing the detection for the three compounds, the next step was the characterization of the sensor array chosen.

### 3.5. Quantitative Analysis: Partial Least Squares (PLS) Regression

Once the qualitative analysis was completed, the data was processed in order to build a model able to quantify each compound individually. As results of the high complexity of the data, it was mandatory to use pretreatments that leads to less noisy and more homogeneous data interpretation. Eventually, the Standard Normal Variate (SNV) [39] method was used as a pretreatment tool and the Partial Least Squares (PLS) technique for the model building. SNV method allowed to reduce the scatter effect of the measurements applying an easy mathematical process; it consists on the subtraction of the measurement mean to the measure and followed by dividing the data by its standard deviation, obtaining in this way a normalized base line for all the samples. A PLS1 approach was next employed, in which one model with single output was built for each compound. The number of the latent variables (LVs) [40] for each model were also optimized to achieve the lowest error possible and to avoid the overfitting. The final PLS models were defined for eight components or latent variables (LVs) for Acetaminophen and 7 LVs for the other two.

**Figure 10:**
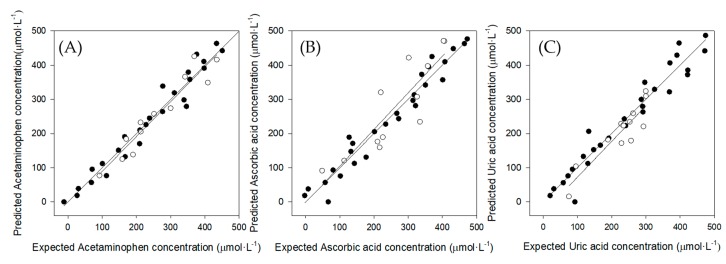
Obtained vs. expected concentrations results plots for the training set (black dots) and the testing set (white dots) for (**A**) Acetaminophen, (**B**) Ascorbic acid and (**C**) Uric acid.

In Figure 10 it can be observed the comparison of the obtained vs. expected results for training sample set (dark dots), for each compound. In the same graph for each compound the testing sample set (white dots) were projected, allowing the visualization of the feasibility of each model.

The comparison general trend was expressed as the linear regression of the comparison line. As it can be observed in Table 3, for all the studied compounds, the y-intercept and slope of the training and testing test regressions include 0 and 1 respectively, taking into account their confidence interval (95%). Regarding the correlation coefficients for all the regressions, they are close to 1. Therefore, this satisfactory trend confirms the potential of the proposed approach.

To evaluate also the fitting degree of the models the NRMSE parameter, Normalized Root Mean Square Error, was calculated according to Equation (1).

(1) $$\mathrm{NRMSE}\;=\;\frac{\sqrt{\frac{\sum_{i}{\left(x_{expected}-x_{predicted}\right)}^{2}}{j\cdot N-1}}}{c_{max}-c_{min}}$$

where *x_expected_* is the theoretical concentration of the sample, *x_predicted_* is the predicted concentration, *j* is the number of analytes considered, *N* the number of samples, *c_max_* is the maximum concentration and *c_min_* is the minimum concentration. All the information about the models are summarized in Table 3.

As can be observed, in practice, the test sample subset had the lowest NRMSE for the Acetaminophen and Uric acid compound, so the predictive capabilities of the models performed in satisfactory way. However, for the Ascorbic acid compound slightly larger values were obtained. Regarding to the correlation coefficients, the better values were observed for the training sample set.

Comparing these results with previous work [41] done in our group employing the same modifiers but with different technology, in this case, the bulk modification of a graphite-epoxy composite electrode (Table 4), we can highlight as a main conclusion that the results obtained are similar, showing a slight improvement in the present report in terms of slope and intercept of the comparison lines. Accordingly, the combination of the screen-printed integrated voltammetry array sensors and chemometric tools allows the possibility to determine and quantify simultaneously a substance in the presence of other ones with overlapping redox potential.

## 4. Conclusions

The presented work reported for a first time in our group the simultaneous voltammetric detection of Acetaminophen, Ascorbic acid and Uric acid combining a multi screen-printed electrode integrated array with advanced chemometrics. This study clearly illustrates one of the capabilities of the biomimetc systems, concretely, ET. The ET strategy allowed the possibility, first to differentiate the compounds, next, to determine and quantify simultaneously a substance in the presence of other ones with overlapping redox potentials.

The samples were analyzed by combining the Cyclic voltammetry (CV) technique for extracting the fingerprint of the individual substances and mixtures, coupled with chemometrics strategies, which permitted the resolution of the overlapping signal and its identification.

The use of Principal Component Analysis (PCA) as qualitative tool was useful to determine the capability of the sensors to distinguish the different compounds under study, while in a further purpose resolution and quantification of ternary mixtures was achieved employing Partial Least Squares Regression (PLS) model.

Therefore, this work demonstrates the advantages of screen-printed integrated electrochemical sensors for on-field analysis results in a promising methodology that could substitute the classical time-consuming, methods. Future works will try to detect these analytes in real pharmaceutical study case.

## Figures and Tables

**Table 1:** Calibration data (*y* vs. *x*) for the separate determination of Acetaminophen, Ascorbic acid and Uric acid employing the integrated sensor array chosen.

Compounds	Graphite	Cobalt (II) Phthalocyanine	Copper Oxide (II)	Prussian Blue
Acetaminophen	*y* = 0.1234*x* + 2.6656 R^2^ = 0.993	*y* = 0.1057*x* + 2.7753 R^2^ = 0.991	*y* = 0.1749*x* − 4.0453 R^2^ = 0.997	*y* = 0.1696*x* + 7.5897 R^2^ = 0.984
Ascorbic Acid	*y* = 0.0398*x* + 6.5307 R^2^ = 0.997	*y* = 0.0311*x* + 8.5678 R^2^ = 0.993	*y* = 0.0372*x* + 3.8868 R^2^ = 0.992	*y* = 0.0214*x* + 9.0457 R^2^ = 0.992
Uric Acid	*y* = 0.1298*x* + 4.6275 R^2^ = 0.999	*y* = 0.0534*x* + 9.3828 R^2^ = 0.996	*y* = 0.1569*x* + 15.231 R^2^ = 0.988	*y* = 0.1074*x* + 7.6534 R^2^ = 0.995

**Table 2:** Reproducibility of construction of each sensor with the results of the relative standard deviation (RSD).

Sensor	RSD%
Graphite/SPCE-Ink	2.9
Cobalt (II) phthalocyanine/SPCE-Ink	7.5
Copper oxide (II)/SPCE-Ink	1.3
Prussian blue/SPCE-Ink	0.8

**Table 3:** Results of the fitted regression curves for obtained vs. expected values, for the training and testing subsets of samples and the three considered compounds (intervals calculated at the 95% confidence level). NRMSE: normalized root mean square error.

Set	Analyte	R^2^	Slope	Intercept (µmol·L^−1^)	NRMSE
	Acetaminophen	0.962	1.00 ± 0.09	0 ± 25	0.90
Training Set	Ascorbic acid	0.955	1.00 ± 0.09	0 ± 25	0.97
(n = 27)	Uric acid	0.940	1.00 ± 0.11	0 ± 31	1.12
	Acetaminophen	0.915	1.02 ± 0.22	−13 ± 28	0.7
Testing Set	Ascorbic acid	0.762	1.07 ± 0.42	−3 ± 54	1.41
(n = 12)	Uric acid	0.829	1.04 ± 0.33	−32 ± 36	0.85

**Table 4:** Results of the fitted regression curves for obtained vs. expected values, for the training and testing subsets of samples and the three considered compounds (intervals calculated at the 95% confidence level) for the previous works [41].

Set	Analyte	R^2^	Slope	Intercept (µmol·L^−1^)
	Acetaminophen	0.968	0.942 ± 0.031	32 ± 21
Training Set	Ascorbic acid	0.947	0.933 ± 0.040	36 ± 25
(n = 33)	Uric acid	0.923	0.873 ± 0.046	58 ± 25
	Acetaminophen	0.848	0.895 ± 0.105	82 ± 71
Testing Set	Ascorbic acid	0.908	0.919 ± 0.081	65 ± 41
(n = 15)	Uric acid	0.753	0.871 ± 0.138	−8 ± 86
